# Model of a sparse encoding neuron

**DOI:** 10.1186/1471-2202-13-S1-P3

**Published:** 2012-07-16

**Authors:** Praveen K Yenduri, Anna C Gilbert, Jun Zhang

**Affiliations:** 1 Department of EECS, University of Michigan, Ann Arbor, MI 48109, USA; 2 Department of Mathematics, University of Michigan, Ann Arbor, MI 48109, USA; 3 Department of Psychology, University of Michigan, Ann Arbor, MI 48109, USA

## 

Neurons as Time Encoding Machines (TEMs) have been proposed to capture the information present in sensory stimuli and to encode it into spike trains [1]. These neurons, however, produce spikes at firing rates above Nyquist, which is usually much higher than the amount of information actually present in stimuli. We propose a low-rate neuron which exploits the sparsity or compressibility present in natural signals to produce spikes at a firing rate proportional to the amount of information present in the signal rather than its duration. We consider the IAF (Integrate-and-Fire) neuron circuit as presented in [1], provide appropriate modifications to convert it into a low-rate encoder and develop an algorithm for reconstructing the input stimulus using Compressive Sampling (CS) techniques. The class of input signals is assumed to be a mixture of periodic waveforms, consistent with the brain mechanism of generating and entraining oscillations at multiple frequencies (S in number) simultaneously. The LowRate IAF neuron circuit uses fixed thresholds (δ) as opposed to random thresholds used in [1]. The randomness in inter-spike-interval exhibited in spike trains is produced by an additional component that switches off the IAF circuit (mimicking the “absolute refractory” period) for a random amount of time (with mean μ) after each spike (see figure 1A). We compare the performance of our LowRate neuron firing at spike-rate K (which is determined by the parameters δ and μ) with IAF neurons in [1] operating at and above Nyquist rate N (>K). Because we inject additive white Gaussian noise into the input signal, we use the traditional measure of signal-to-noise ratio (SNR) as our performance metric. The recovery method developed is a greedy pursuit algorithm similar to the one described in [2]. Figure 1B plots the mean output SNR vs. input SNR for a signal with S = 10 frequencies and sparse-encoding ratio K/N = 0.3052. The LowRate IAF neuron (even when operating at about one third the Nyquist rate in this example) outperforms the IAF neurons operating at and above Nyquist rates. Figure 1C (for signals with S = 60) demonstrates that an increase in sparse-encoding ratio K/N improves the performance of LowRate IAF neuron. We are currently extending this methodology to signals sparse in other domains as well.

**Figure 1:**
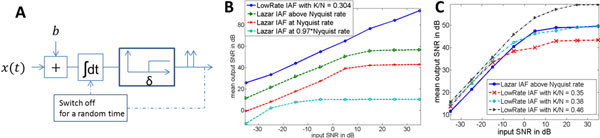
CS IAF neuron **A)** circuit; output SNR vs. input SNR for **B)** signals with S = 10 **C)** signals with S = 60.

## Conclusions

By exploiting sparsity, the LowRate IAF neuron encodes the information present in the input stimulus into spike trains with average firing rate well below Nyquist rate while using the spiking information in a smart manner to improve stimulus recovery.
